# Supplementary material for: A tryparedoxin-coupled biosensor reveals a mitochondrial trypanothione metabolism in trypanosomes
Source: eLife. 2020 Jan 31;9:e53227. doi: 10.7554/eLife.53227 (PMC7046469; doi:10.7554/eLife.53227)
Supplement: Supplementary file 1. [file elife-53227-supp1.docx]

**Key resources table**

| **Reagent type (species) or resource** | **Designation** | **Source or reference** | **Identifiers** | **Additional information** |
| --- | --- | --- | --- | --- |
| Gene (*Trypanosoma brucei brucei*) | *tpx* |  | TriTrypDatabase ID: Tb927.3.3760 |  |
| Cell line (*Trypanosoma brucei brucei*) | WT PC | PMID: 9108552 | WT PC 449 Lister strain 427 | Culture-adapted *T. brucei* strains stably expressing the tetracycline repressor |
| Cell line (*Trypanosoma brucei brucei*) | PC Tpx-roGFP2 | this work |  | PC WT cells which constitutively express cytosolic Tpx-roGFP2 |
| Cell line (*Trypanosoma brucei brucei*) | PC roGFP2-hGrx1 | this work |  | PC WT cells which constitutively express cytosolic roGFP2-hGrx1 |
| Cell line (*Trypanosoma brucei brucei*) | PC roGFP2 | this work |  | PC WT cells which constitutively express cytosolic roGFP2 |
| Cell line (*Trypanosoma brucei brucei*) | PC mito-Tpx-roGFP2 | this work |  | PC WT cells which constitutively express mitochondrial mito-roGFP2-Tpx |
| Cell line (*Trypanosoma brucei brucei*) | PC mito-roGFP2-hGrx1 | this work |  | PC WT cells which constitutively express mitochondrial mito-roGFP2-hGrx1 |
| Cell line (*Trypanosoma brucei brucei*) | PC mito-roGFP2 | this work |  | PC WT cells which constitutively express mitochondrial mito-roGFP2 |
| Cell line (*Trypanosoma brucei brucei*) | PC roGFP2-hGrx1 + Tpx RNAi | this work |  | PC roGFP2-hGrx1 cells that contain a Tet-inducible construct for Tpx RNAi |
| Cell line (*Trypanosoma brucei brucei*) | PC roGFP2 + Tpx RNAi | this work |  | PC roGFP2 cells that contain a Tet-inducible construct for Tpx RNAi |
| Cell line (*Trypanosoma brucei brucei*) | PC mito-roGFP2-hGrx1 + Tpx RNAi | this work |  | PC mito-roGFP2-hGrx1 cells that contain a Tet-inducible construct for Tpx RNAi |
| Cell line (*Trypanosoma brucei brucei*) | PC mito-roGFP2 + Tpx RNAi | this work |  | PC mito-roGFP2 cells that contain a Tet-inducible construct for Tpx RNAi |
| Cell line (*Trypanosoma brucei brucei*) | PC Tpx-roGFP2 + PxI-III RNAi | this work |  | PC Tpx-roGFP2 cells that contain a Tet-inducible construct for PxI-III RNAi |
| Cell line (*Trypanosoma brucei brucei*) | PC roGFP2-hGrx1 + PxI-III RNAi | this work |  | PC roGFP2-hGrx1 cells that contain a Tet-inducible construct for PxI-III RNAi |
| Cell line (*Trypanosoma brucei brucei*) | PC roGFP2 + PxI-III RNAi | this work |  | PC roGFP2 cells that contain a Tet-inducible construct for PxI-III RNAi |
| Cell line (*Trypanosoma brucei brucei*) | PC Tpx-roGFP2 + mPrx RNAi | this work |  | PC Tpx-roGFP2 cells that contain a Tet-inducible construct for mPrx RNAi |
| Cell line (Trypanosoma *brucei brucei*) | PC roGFP2 + mPrx RNAi | this work |  | PC roGFP2 cells that contain a Tet-inducible construct for mPrx RNAi |
| Cell line (*Trypanosoma brucei brucei*) | PC mito-Tpx-roGFP2 + mPrx RNAi | this work |  | PC mito-roGFP2-Tpx cells that contain a Tet-inducible construct for mPrx RNAi |
| Cell line (*Trypanosoma brucei brucei*) | PC mito-roGFP2 + mPrx RNAi | this work |  | PC mito-roGFP2 cells that contain a Tet-inducible construct for mPrx RNAi |
| Antibody | Rabbit anti-Tpx (polyclonal) | PMID: 17040206 |  | WB (1:2000) |
| Antibody | Guinea pig anti-mPrx (polyclonal) | PMID: 29413965 |  | WB: (1:10000) |
| Antibody | HRP-conjugated goat anti-rabbit IgGs (polyclonal) | Thermo Scientific | 31460  RRID: AB_228341 | WB: (1:20000) |
| Antibody | HRP-conjugated donkey anti-guinea pig IgGs (polyclonal) | Merck | AP193P  RRID: AB_92662 | WB: (1:20000) |
| Recombinant DNA reagent | pQE-60_*tpx-rogfp2* | this work |  | Plasmid for expression of recombinant Tpx-roGFP2-His_6_ |
| Recombinant DNA reagent | pQE-60_*hgrx1-rogfp2* | PMID: 18469822 | RRID:Addgene_64799 |  |
| Recombinant DNA reagent | pQE-60_*rogfp2* | Tobias Dick, Heidelberg, Germany | RRID:Addgene_65046 |  |
| Recombinant DNA reagent | pHD1991_*tpx-rogfp2* | this work |  | Plasmid for constitutive expression of cytosolic Tpx-roGFP2 in *T. brucei* |
| Recombinant DNA reagent | pHD1991_*rogfp2-hgrx1* | this work |  | Plasmid for constitutive expression of cytosolic roGFP2-hGrx1 in *T. brucei* |
| Recombinant DNA reagent | pHD1991_*rogfp2* | this work |  | Plasmid for constitutive expression of cytosolic roGFP2 in *T. brucei* |
| Recombinant DNA reagent | pCaSpeR4_*mito-rogfp2-hgrx1* | PMID: 23954927 | RRID:Addgene_65000 |  |
| Recombinant DNA reagent | pHD1991_*mito-rogfp2-tpx* | this work |  | Plasmid for constitutive expression of mitochondrial roGFP2-Tpx in *T. brucei* |
| Recombinant DNA reagent | pHD1991_*mito-rogfp2-hgrx1* | this work |  | Plasmid for constitutive expression of mitochondrial roGFP2-hGrx1 in *T. brucei* |
| Recombinant DNA reagent | pHD1991_*mito-rogfp2* | this work |  | Plasmid for constitutive expression of mitochondrial roGFP2 in *T. brucei* |
| Recombinant DNA reagent | pHD678_*tpx* | this work |  | Plasmid for tetracyclin-inducible RNAi against Tpx |
| Recombinant DNA reagent | pHD678_*pxIII* | PMID: 30047863 |  |  |
| Recombinant DNA reagent | pHD678_*mprx* | Bogacz et al., unpublished |  |  |
| Peptide, recombinant protein | *T. brucei* trypanothione reductase (TR) | PMID: 24788386 |  |  |
| Peptide, recombinant protein | human glutathione reductase (hGR) | PMID: 8097111 |  |  |
| Chemical compound, drug | trypanothione, reduced and oxidized | PMID: 19477177 |  |  |
| Chemical compound, drug | glutathionylspermidine, reduced and oxidized | PMID: 23814051 |  |  |
| Chemical compound, drug | L-glutathione, reduced | Sigma-Aldrich | G4251 |  |
| Chemical compound, drug | L-glutathione, oxidized | Serva | 23130 |  |
| Chemical compound, drug | DL-α-difluoromethyl-ornithine | Cayman Chemicals | 16889 |  |
| Chemical compound, drug | diamide | Sigma-Aldrich | D3648 |  |
| Chemical compound, drug | 1,4-dithiotreitol | biomol | 04010 |  |
| Chemical compound, drug | hydrogen peroxide | Merck | 07209 |  |
