# Supplementary material for: A tryparedoxin-coupled biosensor reveals a mitochondrial trypanothione metabolism in trypanosomes
Source: eLife. 2020 Jan 31;9:e53227. doi: 10.7554/eLife.53227 (PMC7046469; doi:10.7554/eLife.53227)
Supplement: Supplementary file 2. [file elife-53227-supp2.docx]

**Primers generated and used in this work**

| **Name** | **Sequence** | **Additional information** |
| --- | --- | --- |
| GroGFP-Acc-fw | 5’CACAGAATTCATTAAAGAGGAGAAAGGTACCATGGCTCAAGAGTTTGTGAACTG3‘ | Primer for generation of *Acc65*I site in front of *hgrx1* start codon in pQE-60_*hgrx1-rogfp2* |
| GroGFP-Acc-rv | 5‘CAGTTCACAAACTCTTGAGCCATGGTACCTTTCTCCTCTTTAATGAATTCTGTG3‘ | Primer for generation of *Acc65*I site in front of *hgrx1* start codon in pQE-60_*hgrx1-rogfp2* |
| GroGFP-Mlu-fw | 5‘GGAGCTCTGCAGACGCGTGGTGGTTCAGGTGG3‘ | Primer for generation of *Mlu*I site between the *hgrx1* and the linker sequence in pQE-60_*hgrx1-rogfp2* |
| GroGFP-Mlu-rv | 5‘CCACCTGAACCACCACGCGTCTGCAGAGCTCC3‘ | Primer for generation of *Mlu*I site between the *hgrx1* and the linker sequence in pQE-60_*hgrx1-rogfp2* |
| Tpx-Acc-fw | 5‘GATCGGTACCATGTCTGGCCTCGC3‘ | Primer for amplification of *tpx* with 5’*Acc65*I site |
| Tpx-Mlu-rv | 5‘GATCACGCGTGTTGGGCCACGG3‘ | Primer for amplification of *tpx* with 3’*Mlu*I site |
| Tpx-Bam1-fw | 5‘GTGAAGTTTCACTGGGCTCCCTCGTTGGGAAAAC3‘ | Primer to remove first *BamH*I site in the *tpx* coding region |
| Tpx-Bam1-rv | 5‘GTTTTCCCAACGAGGGAGCCCAGTGAAACTTCAC3‘ | Primer to remove first *BamH*I site in the *tpx* coding region |
| Tpx-Bam2-fw | 5‘GTACCCGTGTCATTGAAGATCCCGATGGTGCC3‘ | Primer to remove second *BamH*I site in the *tpx* coding region |
| Tpx-Bam2-rv | 5‘GGCACCATCGGGATCTTCAATGACACGGGTAC3‘ | Primer to remove second *BamH*I site in the *tpx* coding region |
| pHD-roGFP2-fw | 5‘AGTAAGCTTATGGTGAGCAAGGGCGAG3‘ | Primer for amplification of *rogfp2* |
| pHD-Tpx-fw | 5‘GACCAAGCTTATGTCTGGCCTCGC3‘ | Primer for amplification of *tpx-rogfp2* |
| pHD-Grx1-fw | 5‘GGCCAAGCTTATGGCTCAAGAGTTTGTG3‘ | Primer for amplification of *hgrx1-rogfp2* |
| pHD-roGFP2-rv | 5‘GATCGGATCCTTACTTGTACAGCTCGTC3‘ | Primer for amplification of *rogfp2*, *tpx-rogfp2*, and *hgrx1-rogfp2* |
| mito-HindIII-fw | 5‘CATAAGCTTATGTCCGTCCTGACGC3‘ | Primer for amplification of *mito-rogfp2-hgrx1* |
| roGFP2-hGrx1-BamHI-rv | 5‘CGACGGATCCTTAAGCTCCAATCTGC3‘ | Primer for amplification of *mito-rogfp2-hgrx1* |
| Mito -BamHI-fw | 5‘CGTTGCCGCCGGGCTCCGTGAGCAAGG3‘ | Primer to remove *BamH*I site after the mitochondrial targeting sequence in *mito-rogfp2-hgrx1* |
| Mito -BamHI-rv | 5‘CCTTGCTCACGGAGCCCGGCGGCAACG3‘ | Primer to remove *BamH*I site after the mitochondrial targeting sequence in *mito-rogfp2-hgrx1* |
| roGFP2-Tpx-EcoRI-fw | 5‘GATCGAATTCTCTGGCCTCGCCAAG3‘ | Primer for amplification of *tpx* to replace *hgrx1* in *mito-rogfp2-hgrx1* |
| roGFP2-Tpx-BamHI-rv | 5‘GATCGGATCCTCAGTTGGGCCACG3‘ | Primer for amplification of *tpx* to replace *hgrx1* in *mito-rogfp2-hgrx1* |
| Tpx-RNAi-fw_1 | 5‘GATCAAGCTTCTCGTTGGGAAAACTGTG3‘ | Primer for amplification of *tpx* (long fragment) |
| Tpx-RNAi-rv_1 | 5‘GAGCGAATTCTTCCTTCACAGACAGC3‘ | Primer for amplification of *tpx* (long fragment) |
| Tpx-RNAi-fw_2 | 5‘GATCGTTAACCTCGTTGGGAAAACTGTG3‘ | Primer for amplification of *tpx* (short fragment) |
| Tpx-RNAi-rv_2 | 5‘GATCGAATTCGGATCCTCAATGACACG3‘ | Primer for amplification of *tpx* (short fragment) |
